# Generalized lichen nitidus-like eruption following dupilumab initiation

**DOI:** 10.1016/j.jdcr.2026.05.046

**Published:** 2026-05-27

**Authors:** Kyle Mueller, Aleodor A. Andea, Drew Kuraitis

**Affiliations:** a Jacobs School of Medicine and Biomedical Sciences, Buffalo, New York; b Department of Dermatology, Roswell Park Comprehensive Cancer Center, Buffalo, New York; c Department of Pathology, Roswell Park Comprehensive Cancer Center, Buffalo, New York; d Department of Dermatology, Tulane University School of Medicine, New Orleans, Louisiana

**Keywords:** adult lichen nitidus, atopic dermatitis, drug-induced lichen nitidus, dupilumab, dupilumab-associated reaction, generalized lichen nitidus, iatrogenic, lichen nitidus

## Introduction

Lichen nitidus (LN) is an uncommon inflammatory dermatosis that appears as grouped small skin-colored papules, usually affecting children and younger adults, and may have an increased prevalence in Black individuals.^1^ Generally, LN presents as focal and localized papules on a young patient, but LN may rarely present as a generalized eruption, even less commonly seen in adults.^1^, ^2^, ^3^ Histopathologically, LN is characterized by a “claw clutching a ball” pattern comprised of an epidermal collarette surrounding inflammatory infiltrate.^1^^,^^2^ Although the pathophysiologic drivers remain largely unknown, immune-mediated pathways^2^ and genetic factors may be involved.^3^ Dupilumab is a monoclonal antibody that targets interleukin-4 receptor alpha subunit to downregulate the Th2 immune pathway in the treatment of chronic pruritic conditions, such as atopic dermatitis and prurigo nodularis.^4^ Dupilumab has multiple potential cutaneous adverse events, but LN is not regarded as one. Here, we report an adult patient with a generalized LN-like eruption coincident with a single dose of dupilumab treatment for atopic dermatitis.

## Clinical case

**Fig 1:**
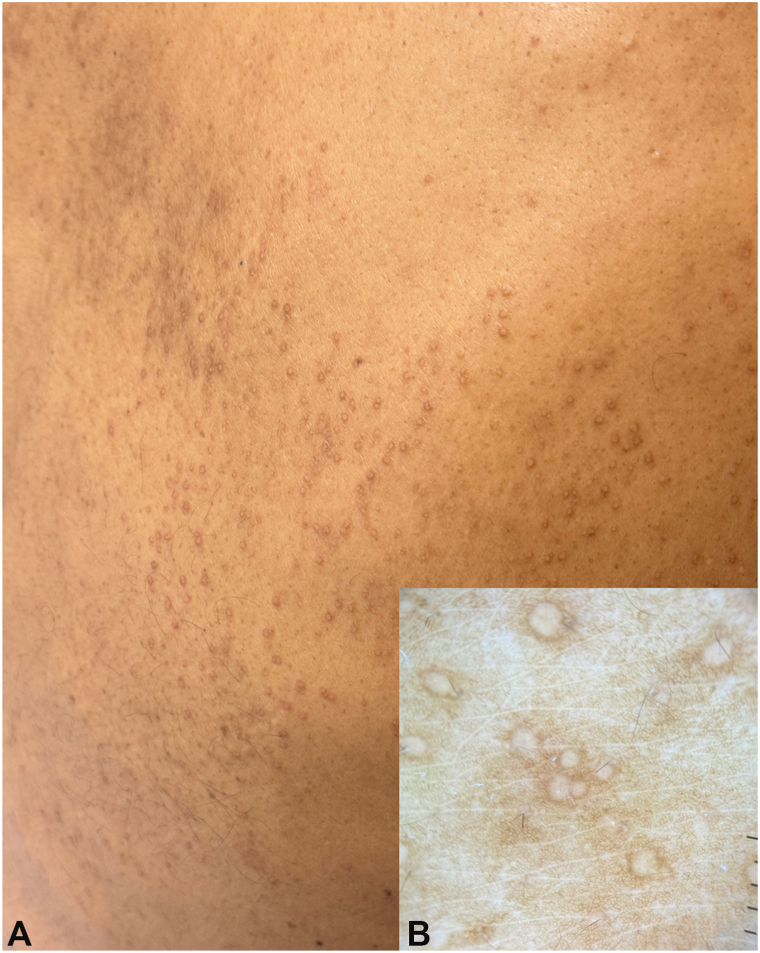
Generalized lichen nitidus. Distinct, skin-colored, flat-topped non-scaling papules, with representative photo showing lesions on upper back **(A)**. Dermoscopy of lesion on the upper back demonstrating monomorphous hypopigmented-to-flesh colored papules with surrounding hyperpigmentation **(B)**.

**Fig 2:**
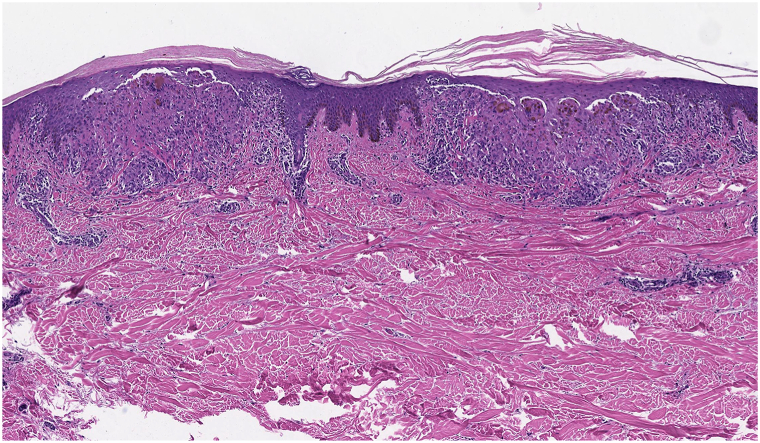
Histopathologic findings of generalized lichen nitidus. Lichenoid dermal inflammatory infiltrates forming small papillary dermal nodules closely abutting the overlying epidermis (A; 20×).

**Fig 3:**
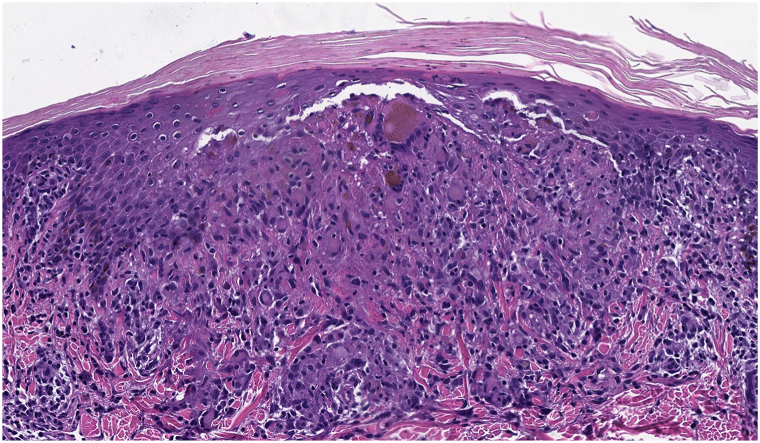
Histopathologic findings of generalized lichen nitidus. The epidermis is remarkable for foci of parakeratosis, vacuolar interface changes with dyskeratosis and focal subepidermal clefting and the lichenoid infiltrate is composed of lymphocytes and histiocytes with a granulomatous appearance (B; 40×).

A 71-year-old Black man presented to our clinic with an abrupt-onset asymptomatic rash to the trunk and extremities. Two weeks prior to this, he was prescribed dupilumab for moderate-to-severe atopic dermatitis and had received a loading dose of 600 mg. Physical examination revealed generalized, noncoalescing skin-colored to hypopigmented, flat-topped papules in a nonfollicular pattern to the back, abdomen, and proximal upper and lower extremities, notably sparing the face, palms, soles and genitalia (Fig 1, *A*), with dermoscopy not revealing any specific structural features regarding the papules, but demonstrating prominent hyperpigmentation to the border of lesions (Fig 1, *B*). Histologic examination showed well-circumscribed, lichenoid dermal inflammatory infiltrates forming small papillary dermal nodules closely abutting the overlying epidermis (Fig 2). The infiltrate was composed of lymphocytes and histiocytes with a granulomatous appearance (Fig 3). The epidermis was remarkable for foci of parakeratosis, vacuolar interface changes with dyskeratosis and focal subepidermal clefting. The rapid onset of the eruption after drug initiation without other known changes suggested a drug-induced eruption, while the pathology was most consistent with LN. Suspecting dupilumab as a potential trigger, his injections were held, and he was started on topical triamcinolone 0.1% ointment. The patient declined further dupilumab treatment, as his atopic dermatitis-associated pruritus had largely resolved after a single loading dose of dupilumab. Over a period of 12 months, his generalized LN slowly resolved.

## Discussion

For a patient with an asymptomatic generalized rash consisting of distinct flat-topped, nonscaling skin-colored papules, the differential diagnosis includes LN. However, our patient’s age and the eruptive and generalized nature of the rash did not align well with the typical patient demographics or clinical presentation of LN. Generalized LN is rare, and even more so in adults. Based on clinical features, the differential also included lichen planus and other lichenoid eruptions, a perforating dermatosis, or papular eczema. After biopsy, the histopathological features of granulomatous changes, inflammatory infiltrate, and the classic epidermal changes into a claw shape reinforced LN as the most likely diagnosis. The rarity of this clinical presentation, its rapid onset after dupilumab initiation, and subsequent clearing with discontinuation suggested a dupilumab-associated eruption. However, given that the eruption took 12 months to resolve after dupilumab discontinuation and that a rechallenge was not completed, we cannot exclude that the LN-like eruption was purely coincidental with dupilumab.

Dupilumab is an anti-interleukin-4 receptor α monoclonal antibody with a variety of reported cutaneous adverse events, including paradoxical Th2-driven reactions, lichenoid eruptions, granulomatosis, and psoriasiform dermatitides.^4^^,^^5^ Infrequent reports describe cases of iatrogenic LN, most associated with immunomodulators such as nivolumab,^6^ adalimumab,^7^ and mogamulizumab and tremelimumab.^8^ Notably, the distribution in all these cases included at least two body regions. To our knowledge, only two prior reports associate dupilumab and LN-type eruptions. One case described a 22-year-old patient with a history of eosinophilic esophagitis who developed generalized LN shortly after receiving a first dose of dupilumab.^9^ Despite discontinuing the drug, the eruption did not resolve after 3 weeks, and dupilumab was then restarted. The second case reported a patient with a history of atopic dermatitis treated with dupilumab who experienced resolution of atopic plaques but worsening of plaques to the palms.^10^ After 18 months of treatment with dupilumab, the patient presented with small papules colocalized with the palmar plaques, which were confirmed to be perforating LN on biopsy. Dupilumab was discontinued and the perforating LN was treated with pimecrolimus cream, and the patient experienced some initial improvement before being lost to follow-up. Together, these cases suggest that dupilumab may shift the immune balance toward pathways permissive of LN-type inflammation.

We speculate that dupilumab therapy contributed to LN-type inflammation in our patient and the prior cases via skewing toward Th1/Th17 cytokine signaling. In the therapeutic blockade of Th2-driven autoinflammation, dupilumab treatment can provoke Th1/Th17-mediated inflammatory pathologies, including lichenoid and granulomatous dermatoses, likely due to compensatory immune shifts.^4^^,^^5^ This dupilumab-associated immune imbalance could plausibly induce the development of LN-like granulomatous changes as seen in our patient and the prior cases.^10^ Further research is needed to characterize the immunologic mechanisms in iatrogenic LN and identify the intermediate drivers of dupilumab-associated LN-like eruptions.

Our case adds a unique clinical picture of a generalized LN-like eruption in an older adult patient to the emerging discussion of cutaneous adverse events associated with dupilumab. Recognizing this possibility may allow for earlier identification in similar cases, with appropriate dermatologic referral and informed discussion with patients regarding risk, monitoring, and management of dupilumab-associated adverse events.

## Conflicts of interest

None disclosed.
